# Non-Invasive Physiological Metrics for Cognitive Load Assessment in Training and Operational Contexts: Signal Processing, Evidence, and Feasibility

**DOI:** 10.3390/s26154848

**Published:** 2026-08-01

**Authors:** Mowffq M. Alsanousi, Vittaldas V. Prabhu

**Affiliations:** 1 Industrial and Manufacturing Engineering Department, Pennsylvania State University, State College, PA 16802, USA; vittal.prabhu@psu.edu; 2 Department of Architectural Engineering and Construction Management, King Fahd University of Petroleum and Minerals, Dhahran 31261, Saudi Arabia

**Keywords:** cognitive load, mental workload, physiological signal processing, wearable sensors, non-invasive monitoring, multimodal fusion, EEG, HRV, electrodermal activity

## Abstract

Cognitive load is a key determinant of performance, safety, and learning in high-stakes environments. Self-report and performance-based methods remain valuable but often miss rapid within-task changes in mental demand, motivating non-invasive physiological sensing for continuous monitoring. The interpretability of these signals depends on their specificity, acquisition quality, signal processing requirements, and real-world feasibility. Integrating physiological mechanism, measurement performance, and operational feasibility, this article reviews non-invasive physiological metrics, including cardiovascular measures (HR/HRV), respiratory metrics, EEG, fNIRS, ocular metrics, and electrodermal activity, for cognitive load assessment across training, simulated, and operational contexts. This structured narrative review synthesized 37 sources published between January 2021 and February 2026, including 25 primary empirical studies and 12 systematic reviews or meta-analyses identified through Google Scholar, PubMed, Scopus, Web of Science, and IEEE Xplore. Among the primary studies, cardiovascular measures were most frequently used (HR/HRV, 16 of 25), followed by EEG (11), ocular metrics (9), electrodermal activity (8), fNIRS (3), and respiratory metrics (3). A consistent trade-off emerged between physiological specificity and ease of deployment. EEG frontal theta showed the most direct and meta-analytically supported link to cortical processing, but it is constrained outside controlled settings by motion artifacts and setup demands. Cardiovascular and electrodermal signals deploy easily through wearables but reflect broader autonomic or sympathetic activation rather than cognitive load specifically. No single metric reviewed here provides both high specificity and strong field readiness. A more defensible approach pairs signals deliberately, based on complementary mechanisms, signal-processing burden, and deployment context, rather than adding sensors indiscriminately. A tiered decision framework and an iterative, context-aware synthesis are proposed to guide metric selection and the interpretation of complementary measurements over time.

## 1. Introduction

Cognitive load refers to the demands placed on an individual’s cognitive system during task performance, including demands generated by task complexity, task design, and the construction of mental representations [1,2]. In high-stakes environments such as aviation, military operations, healthcare, manufacturing, and education, maintaining cognitive demand within operator capacity is essential for reducing errors, preserving safety, and supporting learning [3]. When task demands exceed available cognitive resources, decision-making, response speed, and performance reliability may deteriorate [4,5,6].

Cognitive load is commonly assessed using self-report instruments and performance-based measures [2,7]. Although these methods remain valuable, they are limited by retrospective bias, individual differences in self-awareness, and reduced sensitivity to rapid within-task fluctuations [2,7]. Although performance outcomes such as accuracy and response time may indicate that performance changed, they do not always explain whether the change resulted from cognitive overload, fatigue, stress, or task strategy [3]. These limitations have motivated growing interest in physiological signals that can be recorded continuously and used as temporally sensitive indicators of cognitive state [8,9].

Non-invasive physiological signals provide complementary information because they can be recorded continuously, with limited interruption to task performance. Common signals include heart rate and heart rate variability (HR/HRV), electrodermal activity (EDA), respiratory patterns, electroencephalography (EEG), functional near-infrared spectroscopy (fNIRS), pupillometry, and eye-tracking metrics [8,10,11]. These modalities differ in what they measure: EEG and fNIRS are closer to cortical processing, HR/HRV and EDA reflect broader autonomic regulation, ocular metrics capture attentional and arousal-related responses, and respiratory metrics are affected by both cognitive and non-cognitive factors. Recent multimodal state-estimation frameworks have also shown how physiological signals can be combined with behavioral and subjective indicators to support interpretable real-time human state monitoring in Industry 5.0 contexts [12]. The broader adoption of human-augmentation technologies, including extended reality, collaborative robots, and AI-assisted tools, in operational and training environments further intensifies the need for reliable cognitive load monitoring by introducing new sources of mental demand and human–machine interaction complexity [13].

Several highly relevant reviews have examined physiological approaches to cognitive workload assessment. Some emphasize computational and machine-learning approaches to workload estimation [8], whereas others synthesize physiological metrics used in real-world or operational settings, including a systematic review of neurophysiological measures for mental workload in real-world contexts [9] and an earlier deployment-oriented review designed to guide the selection of physiological workload measures [14]. More recent reviews provide overviews of wearable methods for cognitive workload estimation in human–machine interaction [15] and compare multimodal sensing across both physical and cognitive workload [16]. However, these reviews do not primarily evaluate physiological metrics through an integrated comparison of physiological specificity, signal-processing burden, validity threats, and deployment feasibility across laboratory, training, and operational environments. To address this gap, the present structured narrative review synthesizes recent empirical and review evidence into a context-driven framework for metric selection. By making the trade-offs among physiological specificity, evidence strength, processing burden, confounding, and deployment feasibility explicit, the review can help researchers select appropriate signals during study design, identify appropriately matched sensor combinations, and anticipate the preprocessing and validation requirements of the intended setting.

Despite extensive research on physiological cognitive load assessment, several issues remain only partly resolved. First, the physiological pathways linking cognitive demand to observable signals can be difficult to separate fully from general arousal or stress responses [9]. Second, the empirical evidence differs across modalities; some indicators, such as EEG frontal theta activity, are supported by stronger evidence, whereas others remain mixed or exploratory [17,18]. Third, practical deployment remains constrained by motion artifacts, environmental noise, sensor burden, preprocessing variability, and limited standardization [19,20]. Hence, this narrative review aims to evaluate the physiological metrics used for cognitive load assessment, examining their neurophysiological foundations, measurement performance, and applicability in training and operational contexts. Building on the cross-metric synthesis, the review also outlines an iterative, context-aware framework for interpreting complementary measurements over time.

## 2. Methods

In this review, cognitive load denotes the demand imposed on a person’s limited-capacity cognitive resources during task performance, arising from task complexity, task design, and the mental representations a task requires [1]. It is treated as one component of the broader construct of mental workload, which describes the relationship between imposed task demands and the capacity available to meet them; a recent survey offers an inclusive definition of mental workload spanning these related notions [2]. Cognitive load is distinguished here from mental effort, which refers to the cognitive resources a person actually mobilizes. This distinction is important because a task can impose high cognitive demands while a disengaged operator invests little effort, and the same objective demand can produce different workload depending on individual capacity.

The reviewed primary studies did not share a single operational definition of cognitive load. Instead, they operationalized it through task-demand manipulations, subjective workload or effort scales, performance outcomes, and, in learning contexts, the intrinsic, extraneous, and germane load components of Cognitive Load Theory [1,2]. Examples included difficulty levels, n-back tasks, MATB-II, Tetris difficulty, flight maneuvers, driving scenarios, assembly or surgical complexity, reading or video difficulty, NASA-TLX, RSME, F-ISA, error rate, response time, completion time, and flight-evaluator scores. Because operationalization varied across studies, physiological findings are interpreted in this review in relation to how each study defined and manipulated load, rather than as though a common definition applied.

To make these already-described approaches more explicit, each of the 25 primary empirical studies was reviewed to record how the load construct examined in that study (reported as cognitive load or mental workload) was operationalized through task-demand manipulation, subjective workload or effort assessment, performance outcomes, and/or Cognitive Load Theory components. Because studies could use more than one approach, the categories were non-exclusive. The resulting descriptive counts are reported in the Results section. 

This narrative review focused on non-invasive physiological metrics used for cognitive load assessment, including HR/HRV, respiratory patterns, EEG, fNIRS, pupillometry and eye-tracking measures, and EDA, including GSR and skin conductance measures. This structured narrative review included English-language peer-reviewed journal articles published between January 2021 and February 2026. Primary empirical studies involved human participants in training, simulated, or real-world operational settings, including aviation, military, healthcare, driving, manufacturing, human–robot collaboration, and education.

This recent review window was selected to capture current developments in wearable sensing, real-time physiological monitoring, multimodal classification, and applied cognitive load assessment in Industry 5.0 and related operational domains. Throughout, “signal” means the recorded data stream (for example, ECG, EEG, EDA, or respiration), and “metric” means an indicator derived from it (for example, RMSSD, frontal theta power, Oxy-Hb, pupil diameter, skin conductance level [SCL], or skin conductance response [SCR]).

Relevant literature was identified through searches of Google Scholar, PubMed, Scopus, Web of Science, and IEEE Xplore, supplemented by citation chaining. These search sources were used complementarily because their coverage differs across biomedical, engineering, human-factors, and sensor-related literature. The literature search was iterative and used combinations of cognitive-load, physiological-metric, and context terms. The following search formulation summarizes the main terms used, with wording adjusted across databases: (“cognitive load” OR “mental workload” OR “cognitive workload”) AND (“HRV” OR “ECG” OR “EEG” OR “fNIRS” OR “pupillometry” OR “eye tracking” OR “electrodermal” OR “EDA” OR “GSR” OR “skin conductance” OR “respiration”) AND (“training” OR “simulation” OR “operational” OR “aviation” OR “surgery” OR “military” OR “driving” OR “manufacturing” OR “human-robot collaboration” OR “Industry 5.0” OR “education”).

Primary empirical studies were included if they reported at least one non-invasive physiological metric related to cognitive load, involved human participants in a training, simulated, or operational setting, and validated cognitive load using a workload scale, task-demand manipulation, or performance-based outcome. Review articles were included only if they were systematic reviews or meta-analyses published within the same period and focused on physiological or neurophysiological metrics for cognitive load or mental workload assessment. Studies were excluded if they focused exclusively on clinical diagnosis, used only subjective or performance-based measures without physiological data, addressed contexts unrelated to training or work, targeted constructs other than cognitive load alone (such as stress, fatigue, emotion, or situational awareness), or were not peer-reviewed journal articles.

After the searches were completed, the titles and abstracts of the identified sources were reviewed first, followed by full-text assessment of potentially relevant articles. This screening process resulted in a final corpus of 37 sources published between January 2021 and February 2026, including 25 primary empirical studies and 12 systematic reviews or meta-analyses. The primary studies were used to produce the descriptive sensor-by-domain counts, while the systematic reviews and meta-analyses supported the broader assessment of evidence strength. Additional background, theoretical, methodological, and novelty-positioning references were cited where needed but were not included in the 37-source corpus. Some foundational, definitional, and signal-processing references therefore fell outside the review window.

## 3. Results

### 3.1. Overview of Reviewed Domains and Physiological Signals

Across the reviewed studies, physiological signals were generally interpreted through triangulation rather than in isolation. Changes in physiological measures were compared with task-demand manipulations, subjective workload ratings, performance outcomes, or a combination of these indicators [21,22,23]. This approach is important because most physiological signals are not specific to cognitive load alone and may also reflect stress, fatigue, movement, emotion, or environmental conditions [24,25,26].

The reviewed domains differed in how easily physiological data could be collected and interpreted. Controlled simulators and laboratory settings allowed better control over lighting, movement, and sensor placement, whereas operational and field settings introduced more noise, movement, and practical constraints [18,27,28]. Therefore, the value of each metric depends not only on its sensitivity to cognitive demand but also on whether it can be processed and interpreted reliably in the intended context. The following sections examine these signals in terms of their physiological basis, empirical support, and practical suitability.

### 3.2. Neurophysiological Foundations of Cognitive Load Signals

#### 3.2.1. Cardiovascular Metrics (HR/HRV)

Cognitive demand alters autonomic cardiac regulation, shifting autonomic balance toward sympathetic predominance with concurrent parasympathetic withdrawal, which typically raises heart rate and reduces HRV during elevated mental demand [20,21]. Among the time-domain parameters, the root mean square of successive differences (RMSSD) and the proportion of successive heartbeat intervals differing by more than 50 ms (pNN50) are interpreted as indices of parasympathetic vagal tone, so reductions in these measures are interpreted as diminished vagal influence under increased cognitive demand [20,29]. Cardiovascular dynamics are nonetheless highly susceptible to confounding because physical activity, temperature, respiration, and emotional arousal can each alter autonomic regulation independently of task difficulty [30]. Because peripheral cardiac measures track broad autonomic shifts rather than isolated cognitive processes, they function primarily as indicators of systemic physiological arousal rather than standalone indices of cognitive load [31].

#### 3.2.2. Respiratory Metrics

Breathing is regulated through both voluntary and involuntary pathways, and respiratory dynamics shift with cognitive demand, with elevated respiration rates and more frequent sighing commonly reported as task demand increases [24]. Respiratory measures are, however, strongly affected by non-cognitive factors: speech and other verbal activity directly disrupt breathing patterns, and emotional states can alter respiration regardless of task difficulty [21,24]. These properties make respiratory metrics better suited to cross-validation alongside other signals than to standalone inference; indeed, ocular and respiratory measures together have been reported to track workload more reliably than either alone [24].

#### 3.2.3. Neural Metrics (EEG, fNIRS)

Neural measures offer a more direct window into cognitive processing than peripheral autonomic signals because they index cortical activity rather than downstream systemic responses [17,32,33]. EEG and fNIRS are complementary: EEG records electrical cortical activity, whereas fNIRS measures task-related hemodynamic change through oxygenated and deoxygenated hemoglobin concentrations [32]. EEG activity is usually summarized as the spectral power (the strength of the signal) within standard frequency bands, mainly theta (about 4 to 8 Hz), alpha (about 8 to 12 Hz), and beta (about 13 to 30 Hz). These bands track different processes: frontal theta power rises with working memory load and cognitive control, alpha power tends to fall as a region becomes more engaged (often called alpha suppression), and beta power is linked to active processing and engagement [17]. Under elevated cognitive load, meta-analytic evidence shows increased theta power, particularly frontal, together with reduced alpha power across frontal, central, and parietal regions [17]. A recent multimodal study in a complex, dynamically changing environment likewise reported increased theta power under higher task difficulty, although the alpha response did not follow the meta-analytic pattern [25]. Increased frontal theta power is interpreted as heightened cognitive control and working memory demand, while alpha suppression reflects reduced cortical inhibition and greater engagement with task-relevant information [17]. In parallel, rising demand increases cerebral blood flow and oxygenation in prefrontal regions, typically elevating Oxy-Hb and lowering Deoxy-Hb as metabolic demand rises [32,34]. These hemodynamic changes are frequently localized to the dorsolateral prefrontal cortex and are consistently implicated in working memory and executive control [31]. Neural responses are not uniformly linear, however. At very high workload, cortical activation may decline, possibly reflecting fatigue, disengagement, or resource depletion [25].

#### 3.2.4. Ocular Metrics

Whereas EEG and fNIRS index cortical processing directly, ocular metrics provide a peripheral but neurally grounded window on attentional effort. Pupil dilation is widely used as a non-invasive index of arousal and attentional effort, reflecting autonomic and neurophysiological regulation during demanding tasks [20,21]. Gaze behavior shifts in parallel: fixation durations lengthen when tasks require sustained extraction from specific targets, and eye-movement transitions vary with the integration demands imposed by task complexity [21,35]. Blink rate follows a more complex trajectory, often suppressed during visually demanding tasks yet rebounding as fatigue and cumulative workload mount [21]. This sensitivity carries a cost, as ambient lighting, gaze position, and task-specific visual requirements can substantially affect pupil and eye-movement behavior independently of mental workload [21]. Ocular metrics therefore act as sensitive real-time indices of attentional effort, the reliability of which depends on controlled lighting and systematic baseline normalization.

#### 3.2.5. Electrodermal Activity (EDA)

EDA captures changes in skin electrical conductance driven by eccrine sweat-gland activity and is widely used as an index of sympathetic arousal and general psychophysiological activation [26]. The signal comprises two components: a sustained tonic component, skin conductance level (SCL), reflecting slow baseline activation associated with sustained arousal and engagement, and a transient phasic component, skin conductance response (SCR), consisting of rapid fluctuations to discrete or significant stimuli and abrupt changes in mental demand [26]. Although EDA is sensitive to engagement and arousal, it lacks the specificity to serve as a standalone cognitive load index because sympathetic activation also responds to emotional stress, physical exertion, movement, and ambient temperature [26]. Consistent with this, EDA does not reliably differentiate workload levels in a complex, dynamically changing task environment [25]. EDA is therefore most useful within a multimodal protocol alongside more cognitively specific measures [26]. Table 1 summarizes the neurophysiological mechanisms of the reviewed physiological metrics.

### 3.3. Measurement Performance and Evidence Strength

#### 3.3.1. Cardiovascular and Respiratory Metrics

Evidence for cardiovascular metrics as cognitive load indicators is mixed. Several studies show that higher cognitive demand is associated with increased heart rate and reduced HRV, particularly reduced values of parasympathetic indices such as RMSSD, which are commonly interpreted as reflecting parasympathetic withdrawal during demanding tasks [20,29]. However, the strength of these effects varies across tasks, analysis methods, and comparison conditions [20]. For example, in a large-scale driving simulation (N = 892), higher cognitive load was consistently associated with elevated heart rate and reduced HRV, measured using RMSSD, although average heart rate separated the workload conditions better than the HRV measures did [30]. This suggests that the choice of cardiac metric matters, not only the choice of modality.

The main limitation is that cardiovascular signals reflect broad autonomic activity rather than cognitive load alone. Physical movement, emotional stress, respiration, temperature, and environmental stressors can all affect heart rate and HRV. For example, in military aircrews, hypoxia increased autonomic strain when combined with high cognitive demand [36]. Cardiovascular measures also depend on individual baselines, preprocessing decisions, and recording window length [31]. These issues are especially important in short manual or industrial tasks, where the task may not last long enough to produce a stable HRV response. In one assembly study, RMSSD did not correlate with subjective or performance-based workload; the analyzed HRV sample was small (N = 27, derived from 33 participants after excluding recordings with poor signal quality), and the task duration was relatively short, which may have been insufficient to elicit a stable autonomic shift [22]. More broadly, a single brief recording captures only a narrow time window. In real work, an operator may pass through many task episodes across a shift, and cardiovascular measures can drift with time on task and fatigue, so a value taken early in the day need not match one taken later. This argues for repeated or continuous measurement, with appropriate baseline correction, rather than a single short snapshot.

Evidence for respiratory metrics is more limited but generally suggests that respiration rate increases when cognitive demand rises [24]. However, respiratory signals are strongly affected by non-cognitive factors, especially speech, verbal activity, posture, and movement [21,24]. For this reason, respiratory measures are better used as supporting indicators alongside other physiological signals than as standalone markers of cognitive load.

#### 3.3.2. Neural Metrics

Neural metrics are among the stronger indicators of cognitive load because they are closer to brain activity than peripheral signals such as HRV or EDA. For EEG, frontal theta power was the most consistently supported indicator. Meta-analytic evidence indicates that theta power is significantly affected by cognitive load (g = 0.68, 95% CI [0.41, 0.95]), with frontal theta identified as the best index of cognitive workload, making it one of the most defensible EEG markers for distinguishing high-demand from low-demand conditions [17]. This finding supports the interpretation of frontal theta as a marker of working memory demand and cognitive control. Other EEG bands show less consistent evidence. Beta power also increases under workload in some studies, but unlike frontal theta (which has a well-established link to working memory demand), beta activity is harder to interpret because it can reflect several different processes, including task engagement, working memory maintenance, and top-down attentional control, making it difficult to isolate the cognitive load contribution [17]. Alpha power often decreases as cognitive demand increases, but the effect is weaker and more variable than frontal theta. Alpha changes may also be affected by fatigue during long or repeated tasks, which makes interpretation more difficult [17,25]. Frontal theta can therefore be treated as the strongest EEG indicator, while beta and alpha should be interpreted more cautiously.

The evidence for fNIRS is also supportive. Several studies report increased Oxy-Hb and decreased Deoxy-Hb in prefrontal regions during higher cognitive demand, including surgical training [32,34,37]. This pattern suggests increased blood flow and oxygen use in brain areas involved in working memory and executive control. However, fNIRS responses may not always increase in a simple, linear way. In complex or prolonged tasks, prefrontal activation may decline at very high workload, possibly because of overload, fatigue, or reduced engagement [25]. Despite their sensitivity, EEG and fNIRS remain difficult to use reliably in all settings. EEG is vulnerable to motion artifacts, muscle activity, environmental noise, and preprocessing differences, while fNIRS can be affected by head movement, sensor placement, and participant characteristics, such as dense hair. These issues can reduce signal quality and make the results harder to compare across studies [17,27].

#### 3.3.3. Ocular and Electrodermal Metrics

Among ocular metrics, pupil dilation appears to be the most sensitive indicator of cognitive load. Several studies report that pupil size increases as task complexity rises [20,21], and meta-analytic evidence from educational tasks also supports this pattern, with pupil dilation significantly increased under hard task conditions (d = 0.72, 95% CI [0.37, 1.07], *p* < 0.0001) [38]. In contrast, fixation duration, fixation count, and blink-related measures show less consistent results and appear to depend more strongly on task design, visual demands, and measurement conditions [21,38]. However, pupil dilation must be interpreted carefully because pupil size is strongly affected by ambient lighting, gaze position, screen characteristics, and other visual factors unrelated to cognitive workload [21,35].

EDA is useful as an indicator of arousal and engagement, but it is less specific to cognitive load. In some studies, EDA improved cognitive load prediction when combined with eye movement and cardiac features [23]. However, EDA does not reliably separate workload levels in a complex and dynamically changing task environment [25]. Because EDA is influenced by emotional stress, movement, temperature, and broader sympathetic activation, it is better used as a complementary signal rather than as a standalone measure of cognitive load [26]. Table 2 summarizes the empirical evidence for each physiological metric as a cognitive load indicator.

#### 3.3.4. Multimodal Classification and Signal Fusion

Combining several signals can improve cognitive load classification, but the benefit comes from complementarity, not from the number of sensors. Fusion helps when the combined signals reflect different underlying mechanisms and are disturbed by different confounders so that one signal is informative where another is weak; simply adding more channels that share the same mechanism and the same artifacts adds cost without adding much information [27,40]. Multimodal models that combine neural, ocular, electrodermal, and cardiovascular signals have outperformed single-sensor models in several settings [27,33,40,41]. Direct comparison is still difficult because reported performance depends heavily on the task, the sensor set, the participants, and the validation method [27,33]. Many machine-learning studies also use small datasets, which raises the risk of overfitting and limits the generalizability of the results when validation is not strict [42]. Overall, fusion is a promising direction, but clearer reporting and more comparable validation are needed before strong claims about relative performance can be made [27,42].

Reported accuracy varied widely across the reviewed studies, from modest HR/HRV-only results in short flight tasks to high accuracy in multimodal aerospace models. Using multimodal physiological signal fusion, one cross-modal aerospace framework combining electromyography, electrooculography, and electrodermal activity reached an AUC of 0.97 (F1 = 94.55) for predicting flight task difficulty, which improved to an AUC of 0.998 (F1 = 97.95) with an enhanced cross-modal transformer network incorporating eye-tracking features [29,40]. These numbers should be read with care because the studies differ in what they classified, the number of workload levels they used, how they validated, and what sensors and tasks were involved. Many used laboratory or simulator settings with relatively small samples, often only a few dozen participants, frequently students or trainees, so the reported accuracy may reflect best-case controlled conditions rather than field performance [42]. Fully operational field evaluations were rare. Future studies should report not only accuracy but also the validation design, how participants were separated between training and testing, how artifacts were handled, and how well models transferred across sessions and users.

### 3.4. Operational Applicability and Wearable Deployment

#### 3.4.1. Wearable Sensor Technology and Real-Time Monitoring

Advances in miniaturized, low-power wearable sensors have enabled continuous, non-invasive, real-time physiological monitoring across diverse operational settings [27]. Portable systems can capture multiple biosignals, including electrocardiography, EDA, respiration, eye-related measures, and increasingly, EEG, while preserving greater mobility than laboratory equipment [27,43]. Consumer-grade wearable EEG has shown growing methodological viability outside the laboratory: a real-world benchmarking study found that consumer headsets can yield usable signals for exploratory cognitive-state monitoring, although signal quality and neurometric stability remained below that of research-grade systems during naturalistic tasks [18]. Similar viability has been reported for mobile EEG in driving, where new sensor systems captured attention-related neuromarkers across automated and manual conditions, although much of this validation has targeted adjacent constructs such as vigilance and fatigue rather than cognitive load directly [44]. Deployment nonetheless involves trade-offs among portability, comfort, and signal quality, and wearable platforms generally provide lower fidelity than laboratory-grade systems [27]. Real-world monitoring is further affected by motion artifacts, variable sensor-to-skin contact, and environmental conditions that degrade signal integrity over extended use [18,27], while battery life and power management remain practical constraints [27].

#### 3.4.2. Signal Quality, Artifacts, and Standardization

Physiological signal quality in operational settings is often degraded by intrinsic and extrinsic noise, including body motion, facial muscle activity, environmental interference, and inconsistent sensor-to-skin contact [27,28]. Although all modalities are affected, EEG is especially vulnerable because movement artifacts overlap with workload-relevant frequency bands [28]. Optical fNIRS may also be compromised when dense hair interferes with optode contact [27]. Reliability is further reduced because motion artifacts and different preprocessing choices make studies harder to compare, with differences in filtering, artifact-rejection criteria, feature-extraction windows, and baseline normalization limiting cross-study comparability and reproducibility [31,33]. Although some standardization exists, such as the International 10–20 system for EEG electrode placement, broader implementation remains inconsistent, particularly for multimodal synchronization, preprocessing, and reporting in applied settings [31,33].

#### 3.4.3. Signal Processing Implications for Wearable Cognitive Load Assessment

Physiological sensors usually record continuous time-series signals that must be processed before they can be interpreted as cognitive load indicators. The typical pipeline includes artifact removal, noise reduction, feature extraction, and validation against workload labels or task conditions. For example, studies may extract RMSSD from HRV, frontal theta from EEG, Oxy-Hb from fNIRS, pupil diameter from ocular data, SCL/SCR from EDA, or respiration rate from respiratory signals. These features are then compared across workload conditions or used as inputs to classification models [40,42]. Reported accuracy therefore reflects not only the sensor itself but also preprocessing choices, feature definitions, labeling strategy, and validation design [27,33].

Established methods can mitigate rather than merely flag these artifacts, and several were used across the reviewed studies. For EEG, independent component analysis (ICA) separates multichannel EEG recordings into statistically independent components, allowing ocular and muscle-related artifacts to be identified and removed. However, conventional ICA may require manual component selection and can be difficult to apply in real time, whereas adaptive filtering can attenuate motion artifacts during ambulant recording [28]. For fNIRS, motion correction and baseline normalization are commonly applied to separate task-related hemodynamic change from movement and systemic noise [45]. Cardiac processing depends on reliable beat detection and correction of ectopic beats before HRV features such as RMSSD are computed over appropriate windows [20,30]. Ocular processing typically requires blink interpolation, baseline correction, and control of luminance and gaze position [20,41], and electrodermal analysis separates the signal into tonic (skin conductance level) and phasic (skin conductance response) components [9,26]. Because aggressive correction can also remove workload-relevant signal, these choices should be reported and, where possible, validated rather than applied blindly.

Each modality also has its own processing challenges. HR/HRV requires reliable beat detection and appropriate window lengths, EEG requires artifact removal and spectral analysis, fNIRS requires correction for motion and vascular noise, ocular metrics require lighting and gaze-position control, EDA requires separation of tonic and phasic components, and respiratory measures require attention to speech, posture, and movement [27,28]. These issues are not minor technical details, because they directly affect whether a signal remains meaningful in real settings. For this reason, wearable cognitive-load systems should be evaluated not only by classification accuracy but also by signal quality, preprocessing transparency, feature reproducibility, synchronization, and validation under realistic deployment conditions [27]. The modalities also differ in temporal continuity: cardiac measures such as ECG provide a continuous beat-to-beat stream, whereas pupillometry and other ocular measures are effectively sampled and are interrupted by blinks and off-target gaze, which reduces the amount of usable data and complicates alignment with other channels [21].

#### 3.4.4. Application Across Training and Operational Domains

In aviation, physiological metrics such as EEG, HRV, and fNIRS are widely used in simulator-based studies; HRV has, for instance, distinguished cognitive load during demanding flight maneuvers, such as turning [46], whereas implementation in actual flight remains limited by motion, equipment, and cockpit constraints [32]. In military contexts, monitoring must be rugged and robust under field conditions; controlled work with military aircrews has characterized autonomic responses to combined cognitive and environmental stressors, but field-grade real-time assessment remains constrained by wearable signal-quality limits [18,36]. In healthcare and surgical training, neurophysiological and ocular sensing may be constrained by sterility, comfort, and dexterity requirements; fNIRS has, nonetheless, quantified surgeons’ prefrontal workload across task complexity [37], which is consistent with systematic review evidence that fNIRS is increasingly applied to occupational workload [31,45].

In manufacturing and Industry 5.0 environments, wearable EEG, cardiac, and electrodermal sensors are increasingly used to monitor operator workload during human–robot collaboration [27,43], a trend confirmed by a comprehensive review of cognitive-workload methods in manufacturing [47]. EEG has quantified the elevated workload of task switching among assembly workers [48]; eye tracking has indexed performance and workload in VR-based telerobotic operation [49]; and preliminary evidence from a medium-scale enterprise suggests that personalized models accounting for individual baseline differences may improve measurement and support workforce allocation [50]. In driving, mobile EEG and ocular measures track attention- and workload-related states across automated and manual operations [21,44]. In education, physiological monitoring is attracting growing interest for adaptive learning: EEG-derived measures have tracked real-time cognitive load during video-based instruction [51], eye-tracking and electrodermal measures have indexed load in multimedia and reading tasks [23,35], and systematic reviews have documented the expanding use of EEG and eye-tracking in 3-D learning environments [52] and of physiological methods in augmented-reality learning [53], although real-time integration into instructional systems remains less established than in simulation-based operational domains. Table 3 reports the descriptive counts of the 25 primary empirical studies by sensor class and domain.

Across the 25 primary empirical studies, task-demand manipulation was used in 21 studies, subjective workload or effort assessment in 18, and performance outcomes in 16 (Table 4). Three studies explicitly operationalized intrinsic, extraneous, and/or germane components of Cognitive Load Theory [23,35,51]. Because many studies combined more than one approach, the category counts exceed 25. This distribution makes the operational variation already described in the Methods more explicit and shows that physiological indicators were commonly interpreted alongside task conditions, subjective assessments, performance outcomes, or combinations of these approaches.

### 3.5. Cross-Metric Synthesis

#### 3.5.1. Physiological Specificity Versus Deployment Feasibility

**Figure 1:**
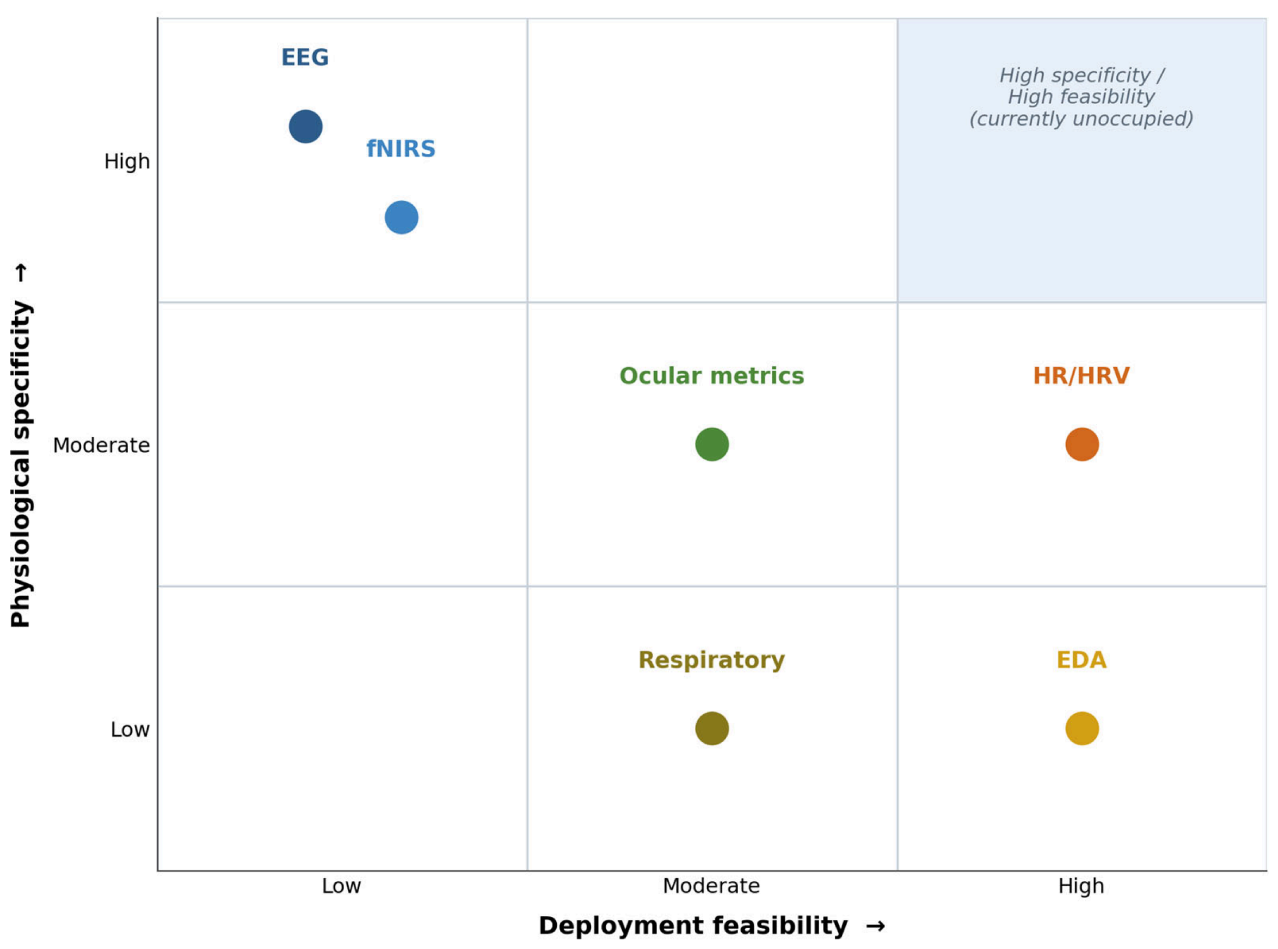
Physiological specificity versus deployment feasibility of non-invasive physiological metrics. Each axis is divided into Low, Moderate, and High bands, and each metric is placed in the cell matching its physiological-specificity and deployment-feasibility ratings; the placements are ordinal, not measured coordinates. Arrows indicate the direction of increase on each axis, the marker colors distinguish the metrics and carry no quantitative meaning, and the shaded upper-right cell marks the high-specificity, high-feasibility combination that no current metric occupies.

A systematic trade-off separates physiological specificity from deployment feasibility across the metrics examined here. Neural measures provide the most direct view of cortical processing but remain technically demanding outside controlled settings, whereas cardiovascular and electrodermal signals deploy more easily, yet offer lower interpretive specificity [21,27]. Ocular metrics occupy an intermediate position, sensitive to attentional change but dependent on controlled visual conditions, particularly stable lighting [21]. Figure 1 positions each modality according to its proximity to cognitive processing and its practical readiness for training or operational use. EEG and fNIRS occupy the high-specificity, lower-feasibility region, while HR/HRV and EDA occupy the higher-feasibility, lower-specificity region; the empty high-specificity, high-feasibility cell captures the central challenge that none of the current metrics delivers both.

#### 3.5.2. Metric Performance Profile Across Evaluative Dimensions

**Figure 2:**
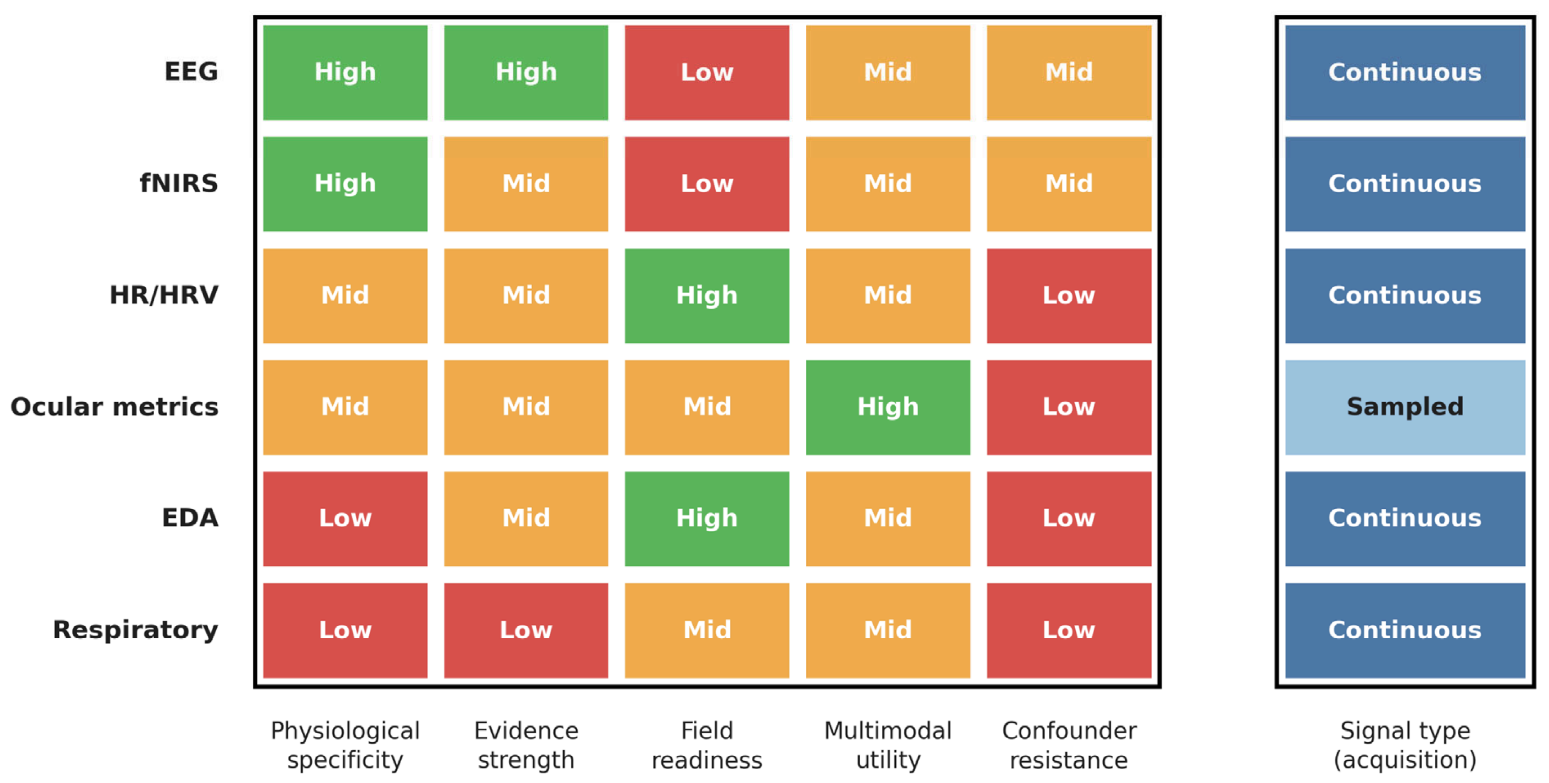
Performance profile of each physiological metric across five evaluative dimensions (Low, Mid, High) and signal type (continuous versus sampled). This profile is context-driven, interpretive metric-selection guidance derived from the qualitative synthesis, not a measured performance ranking.

Figure 2 compares the metrics across five evaluative dimensions (physiological specificity, evidence strength, field readiness, multimodal utility, and confounder resistance), each rated on a three-point interpretive scale (Low, Mid, High) based on the evidence presented in Section 3.3, Section 3.4 and Section 3.5, together with a signal-type descriptor that distinguishes continuously acquired signals from intermittently sampled ones. No metric performs strongly across all dimensions. EEG leads in specificity and evidence strength but is limited by artifact susceptibility and field-readiness constraints, HR/HRV and EDA are more deployable but less specific, ocular metrics, especially pupillometry, offer strong attentional sensitivity under controlled visual conditions, and respiratory metrics remain the least reliable as standalone indicators. This asymmetry supports the deliberate pairing of complementary signals rather than simply adding channels.

## 4. Discussion

The main pattern across the reviewed evidence is that cognitive load monitoring is constrained by a persistent trade-off between physiological specificity and ease of deployment. Signals closer to cortical processing, particularly EEG and fNIRS, provide stronger mechanistic interpretability but remain difficult to deploy reliably in dynamic environments, whereas wearable autonomic signals such as HR/HRV and EDA are easier to record continuously but less specific to cognitive load, being influenced by stress, movement, temperature, and other non-cognitive factors [21,27]. The practical implication is that the goal of cognitive load sensing should be the design of context-sensitive multimodal systems with transparent preprocessing and validation rather than the search for a single optimal metric.

Physiological cognitive load assessment is therefore as much a signal processing and deployment problem as a psychophysiological one. The same signal can yield different interpretations depending on sensor placement, sampling rate, artifact correction, feature extraction window, baseline normalization, and validation design. These factors weigh especially heavily on wearable systems, where movement, sensor–skin contact, environmental variation, and battery constraints shape data quality and continuity [18,27]. High classification accuracy in controlled studies should therefore not be read as field readiness because reported performance often reflects controlled-condition ceilings rather than operational benchmarks [42]. Since standardization is increasingly important due to the rapid growth of applied sensing research, future systems should report the full processing chain, including acquisition settings, preprocessing decisions, feature definitions, multimodal synchronization, and validation strategy [33].

Because the metrics differ in physiological sensitivity, artifact susceptibility, and deployment burden, sensor selection is better treated as a pairing problem than as a question of how many channels to add. The aim is to combine physiologically complementary signals whose artifacts and confounders do not overlap. Adding more sensors that share the same mechanism and the same confounders increases cost and preprocessing burden without adding much independent information. EEG and fNIRS offer complementary electrical and hemodynamic views of cortical activity in controlled settings [34], while HR/HRV and ocular metrics, especially pupillometry, combine autonomic and attentional indicators with a comparatively lower setup burden in semi-controlled training environments [20,21]. EDA can add an arousal-sensitive channel but should not be interpreted as a standalone cognitive load measure [26], and respiratory metrics are better treated as supporting signals unless speech, posture, and movement are tightly controlled [24]. Reliable interpretation under real-world conditions may thus require converging evidence from several complementary physiological channels rather than relying on classification accuracy alone.

Since the choice of processing and modeling approach shapes both accuracy and interpretability, it is useful first to summarize how the reviewed studies turn raw signals into load estimates (Table 5). Approaches range from single-metric statistical comparison through classical machine learning and neural networks to multimodal fusion, temporal or state-based models, and personalized or baseline-normalized models. Each suits a different combination of data availability, interpretability needs, and deployment context.

These findings can be translated into a practical deployment readiness framework (Table 6). The goal is not to rank metrics universally but to guide metric selection based on context. In field settings, HR/HRV is among the most practical options because it is wearable and easy to collect, although it remains sensitive to general arousal and index choice [30,36]. EDA can also be deployed relatively easily, but it is best treated as an arousal-support channel rather than a standalone cognitive-load metric [26]. In semi-controlled training settings, HR/HRV can be paired with portable EEG or ocular metrics, especially pupillometry, when setup time, movement, and lighting are manageable [18,20,38]. In controlled laboratory or simulator settings, EEG frontal theta and fNIRS are more suitable because they provide stronger physiological specificity but require careful setup and preprocessing [17,32,45]. Respiratory measures are best treated as supporting signals because they are strongly affected by speech, posture, movement, and environmental factors [21,24].

Building on these findings, cognitive load monitoring may be understood as an iterative estimation process rather than as a direct reading from any single sensor. The reviewed literature supports combining task-demand and contextual information with physiological signals collected over time, behavioral or performance information, and, where available, subjective indicators [12,22]. Individual baselines should also be considered because the same task may produce different physiological and perceived workload responses across operators [50].

**Figure 3:**
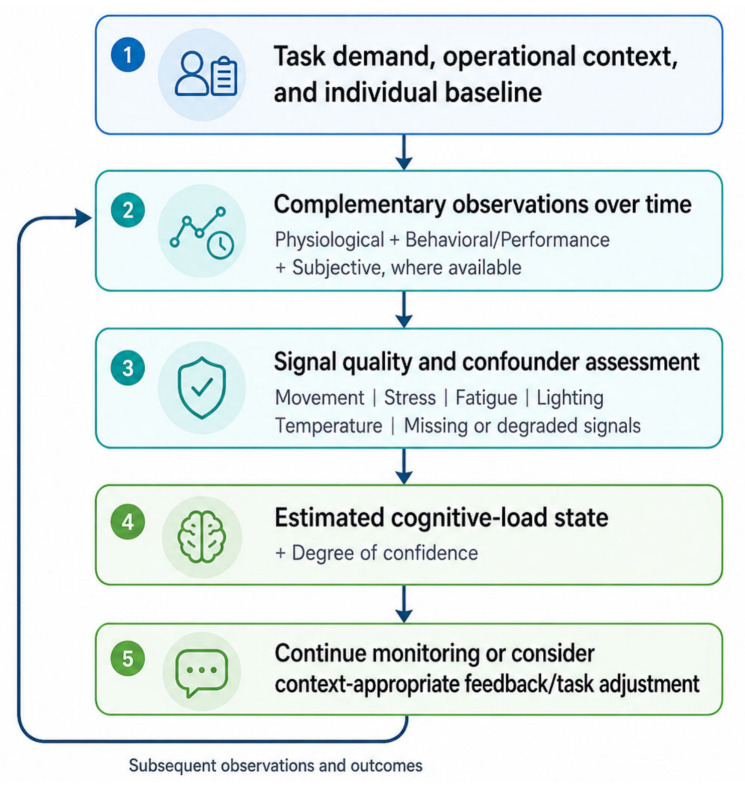
Proposed iterative and context-aware framework for cognitive load monitoring. Task demand, operational context, and individual baseline guide the interpretation of complementary physiological, behavioral or performance, and subjective observations collected over time. Signal quality and plausible confounders are evaluated before the cognitive-load state and its associated degree of confidence are updated. The resulting estimate may support continued monitoring or context-appropriate feedback or task adjustment, with subsequent observations informing the next assessment cycle. The framework represents the authors’ synthesis of the evidence reviewed in this article.

Before the estimate is updated, signal quality and plausible confounders should be evaluated. Movement, stress, fatigue, lighting, and temperature may alter one or more channels independently of cognitive demand [21,25,27]. Missing or degraded signals, inconsistent sensor contact, and other acquisition problems may further reduce reliability [18,27,28]. The resulting output should therefore be interpreted as an estimated cognitive-load state with an associated degree of confidence rather than as a direct measurement of the construct. When evidence remains weak or inconsistent, continued monitoring may be more appropriate than immediate adaptation. Where complementary indicators converge over time, the estimate could eventually support context-appropriate feedback or task adjustment, although prospective validation would still be required [43,51]. Figure 3 summarizes this proposed cycle.

Emerging wearable systems make this approach increasingly practicable because cardiovascular, electrodermal, respiratory, ocular, and some neural signals can be collected repeatedly or continuously in training and operational settings [18,27,43]. The main strengths of the proposed framework are its use of temporal continuity, complementary evidence, contextual interpretation, individual baselines, and explicit uncertainty. Its main limitations include calibration burden, multimodal synchronization, artifact-management requirements, incomplete separation from overlapping states, and the need for validation across participants, sessions, and operational contexts before cognitive-load estimates are used to modify training or task demands [33,42,50].

This article has several strengths. First, it evaluates the reviewed metrics from three connected perspectives: physiological mechanism, empirical evidence, and operational feasibility. This avoids judging metrics by classification accuracy alone and provides a more balanced assessment of their practical value. Second, it compares six major non-invasive signal classes across training, simulated, and operational contexts, making the trade-off between physiological specificity and deployment readiness explicit. Third, it gives specific attention to signal processing and implementation issues, including artifact susceptibility, preprocessing variability, baseline normalization, feature extraction, and multimodal synchronization. Lastly, the review translates the evidence into deployment-oriented guidance for selecting metrics and interpreting complementary measurements over time, rather than assuming that one physiological signal is universally optimal.

Several limitations should be acknowledged. Because this review was designed as a structured narrative synthesis rather than a systematic review or meta-analysis, it does not claim PRISMA compliance, formal risk-of-bias assessment, or pooled effect-size estimation across metrics. The descriptive counts in Table 3 indicate study coverage and should not be interpreted as evidence quality scores. Direct comparison across studies remains difficult because the literature varies in task design, participant characteristics, sensor configuration, preprocessing choices, validation methods, and exposure to confounds such as stress, movement, environmental conditions, speech, and individual baseline differences. Finally, because many studies were conducted in laboratory or simulator settings, further field validation is needed before some metrics can be considered ready for operational deployment.

Future research should prioritize moving physiological cognitive load assessment into realistic training and operational settings. Three directions are especially important. First, the field needs shared protocols for signal acquisition, synchronization, preprocessing, feature extraction, and reporting so that the results can be compared more reliably across studies [27,33]. Second, wearable systems should be evaluated under realistic sources of noise, including movement, variable sensor contact, environmental change, fatigue, and prolonged use [18,27,28]. Third, computational models should be validated across participants, sessions, and contexts, with an emphasis on interpretability, personalization, and robustness rather than accuracy alone [42,50].

Future work may also explore closed-loop adaptive systems that adjust task demands or training feedback in real time. However, these systems will require clearer methods for separating cognitive load from overlapping states, such as stress, physical fatigue, and emotional arousal [25,26]. As continuous biometric monitoring becomes more common in workplace and training environments, future deployments should also consider privacy, ethical use, data ownership, and the long-term acceptability of prolonged sensor use. These priorities are especially important for manufacturing, education, and Industry 5.0 human–robot collaboration, where applied evidence remains comparatively limited [43,47].

A sensing channel not used in the reviewed studies may also be worth future exploration. Beyond the reviewed corpus, acoustic and prosodic features of speech, such as fundamental frequency and intensity, may vary with cognitive load, although these effects overlap with those of stress and emotion [54]. Speech-based estimation has been reported in operational and training-like settings, for example from spoken commands to simulated aircraft [55], and voice combined with cardiovascular measures may improve workload assessment [56]. Because speech is also shaped by accent, language background, speaking style, and recording quality, and because speaking can itself alter the measured state, and because audio is identifiable, any such use would require validation against established measures and careful attention to privacy and consent.

## 5. Conclusions

Non-invasive physiological metrics appear promising for cognitive load assessment, but none of the signals reviewed here offer both high cognitive specificity and strong field readiness. Neural measures (EEG, fNIRS) sit closest to cortical processing yet remain constrained by setup and artifact demands, while cardiovascular, electrodermal, ocular, and respiratory measures deploy more easily but are less specific. Robust monitoring across training and operational contexts therefore depends less on adding sensors and more on deliberately pairing signals with complementary mechanisms and confounders that do not overlap. These signals should be interpreted as converging, context-dependent evidence that is updated over time, rather than as direct readings of cognitive load. Achieving this in the field will require standardized acquisition, preprocessing, and validation protocols, along with field testing that moves physiological monitoring from controlled studies toward reliable real-world deployment.

## Figures and Tables

**Table 1:** Neurophysiological mechanisms of the reviewed physiological metrics.

Metric	Physiological Pathway	Key Indicators Under Load	Primary Confounders	Specificity
HR/HRV	Autonomic shift toward sympathetic predominance with parasympathetic withdrawal, reducing vagal tone	Elevated HR; suppressed RMSSD and pNN50	Physical exertion, emotional arousal, respiration	Moderate
Respiratory	Voluntary and involuntary breathing regulation responding to metabolic and attentional demand	Elevated respiration rate; increased sighing	Speech/verbal activity, emotion, movement	Low
EEG	Cortical electrical oscillations reflecting executive control and working memory mobilization	Enhanced frontal theta; attenuated alpha power	Motion and muscle artifacts; mental fatigue	High
fNIRS	Rising metabolic demand increases prefrontal cerebral blood flow and oxygenation	Prefrontal Oxy-Hb elevation; Deoxy-Hb reduction	Head movement, dense hair, vascular noise	High
Ocular metrics	Autonomic and neurophysiological regulation of pupil size and gaze behavior during attentional effort	Increased pupil diameter; longer fixations; suppressed blink rate	Ambient lighting, gaze position, visual demands	Moderate
EDA	Sympathetic drive of eccrine sweat secretion producing tonic (SCL) and phasic (SCR) responses	Elevated SCL; increased SCR amplitude	Emotional stress, exertion, temperature	Low

Specificity reflects the degree to which a metric is mechanistically proximal to cognitive processing rather than primarily reflecting broad autonomic arousal, stress, physical exertion, or environmental conditions. The rating is comparative; no physiological signal is exclusive to cognitive load.

**Table 2:** Empirical evidence summary for each physiological metric as a cognitive load indicator.

Metric	Evidence Strength	Key Indicator	Representative Studies	Main Validity Threat
HR/HRV	Mixed	RMSSD	[22,30,36]	Inter-individual baseline variability and sensitivity to non-cognitive stressors reduce cross-study consistency
Respiratory	Weak	Respiration rate	[21,24]	Limited primary studies; high susceptibility to speech, vocalization, and movement confounds
EEG	Strong	Frontal theta power	[17,25,39]	Motion and muscle artifacts; fatigue-related spectral change during extended tasks
fNIRS	Moderate	Prefrontal Oxy-Hb	[32,34,37]	Non-linear activation at extreme workload limits sensitivity at high demand
Ocular metrics	Moderate	Pupil diameter (best-supported ocular indicator)	[20,21,38]	Ambient lighting and gaze position override cognitive signals without controlled conditions
EDA	Mixed	SCR/SCL	[23,25,26]	Sensitive to arousal/engagement but limited in isolating cognitive load from emotion, exertion, and environment

Evidence-strength ratings (Strong, Moderate, Mixed, Weak) represent qualitative synthesis judgments based on consistency, replication, domain coverage, systematic-review or meta-analytic support, and susceptibility to confounds. They do not represent pooled quantitative effect estimates.

**Table 3:** Descriptive counts of the 25 primary empirical studies by sensor class and domain.

Metric	Aviation	Military	Healthcare	Manufacturing/HRC	Driving	Education	Controlled Lab	Naturalistic/Mixed	Total
HR/HRV	3	1	0	4	4	1	3	0	16
EEG	2	0	0	2	3	1	2	1	11
fNIRS	1	0	1	0	0	0	1	0	3
Ocular metrics	1	0	0	2	3	2	1	0	9
EDA/GSR	1	0	0	1	2	1	3	0	8
Respiratory	0	0	0	1	1	0	1	0	3

Counts indicate the number of primary empirical studies using each sensor class within each domain (*n* = 25). Multimodal studies contribute to more than one sensor row and may therefore appear in multiple cells. Systematic reviews and meta-analyses are not counted here. These counts describe study coverage and are not a measure of evidence quality or pooled effect size. Ocular metrics include pupillometry, eye tracking, gaze behavior, fixation and saccade measures, blink or eye-closure measures such as PERCLOS, and EOG-derived eye-movement features.

**Table 4:** Non-exclusive approaches used to operationalize cognitive load or mental workload across the 25 primary empirical studies.

Operationalization Approach	Description	Common Examples	Number of Studies	Study References
Task-demand manipulation	Cognitive or mental workload was represented through deliberately varied task difficulty, complexity, demand level, control or automation mode, or experimentally defined operational conditions	Difficulty levels, n-back tasks, MATB-II, Tetris difficulty, flight maneuvers, driving scenarios, assembly or surgical complexity, reading or video difficulty	21	[18,20,21,23,24,25,26,28,30,35,36,37,39,40,41,43,44,46,49,50,51]
Subjective workload or effort assessment	Participants directly reported perceived workload, mental demand, effort, or related experiences	NASA-TLX, RSME, F-ISA, perceived workload and mental-effort ratings	18	[18,22,23,24,25,26,28,30,34,35,39,41,43,44,48,49,50,51]
Performance outcomes	Workload was interpreted or validated using task-performance measures	Accuracy, error rate, response time, completion time, lane deviation, hazard detection, flight-evaluator scores	16	[20,22,23,24,25,26,29,34,35,37,39,40,41,44,49,50]
Cognitive Load Theory components	Intrinsic, extraneous, and/or germane cognitive load was explicitly defined, measured, predicted, or analyzed	Intrinsic, extraneous, and germane load ratings or predictions	3	[23,35,51]

The categories were non-exclusive; therefore, studies using multiple operationalization approaches were counted in each applicable row, and the totals exceed 25.

**Table 5:** Common processing and modeling approaches for physiological cognitive load assessment.

Approach	Typical Use	Strengths	Limitations	When It Is Appropriate
Single-metric statistical comparison	Testing whether one metric differs across load levels	Simple, transparent, easy to interpret	Misses interactions; sensitive to confounders	Early stage or mechanism-focused studies with one signal [24,30]
Classical machine learning (SVM, random forest)	Classifying load from hand-crafted features	Works with small samples; interpretable features	Needs manual feature design; limited temporal modeling	Moderate datasets with well-understood features [42]
Neural networks (CNN, LSTM)	Learning features and patterns directly from signals	Captures complex, non-linear patterns	Needs large datasets; low interpretability; overfitting risk	Larger datasets where accuracy outweighs transparency [40,42]
Multimodal fusion	Combining complementary signals	Higher accuracy when signals differ in mechanism	No gain (and added cost) if signals are redundant	When channels have different mechanisms and confounders [27,40]
Temporal/state models	Tracking how load changes over time	Models sequence and transitions, not just snapshots	More complex; assumptions may not hold	Continuous monitoring and tracking change over time [33]
Personalized/baseline-normalized models	Adapting to individual baselines	Reduces inter-individual variability	Needs per-person calibration data	Settings with large individual differences [50]

Approaches are illustrative of the ways in which reviewed studies convert raw physiological signals into cognitive-load indicators; this list is not an exhaustive machine-learning taxonomy.

**Table 6:** Practical deployment-readiness framework for non-invasive cognitive load monitoring.

Tier	Best-Fit Context	Suggested Use	Main Caution
Field-ready	Operational or mobile settings	HR/HRV, with EDA as an optional arousal-support channel, preferably combined with behavioral or contextual data	Easy to collect but reflects general arousal or sympathetic activation, not cognitive load alone
Emerging	Training facilities and simulators	HR/HRV + portable EEG or pupillometry	Requires artifact control, baseline normalization, and lighting control
Research/laboratory	Controlled labs and high-fidelity simulators	EEG frontal theta + fNIRS	Stronger specificity but higher setup and preprocessing burden
Adjunct	Any context as a supporting channel	EDA and/or respiratory measures as supporting channels	Useful for arousal and context, but weak as standalone cognitive-load indicators

The tiers are intended as practical guidance rather than fixed rankings, since metric suitability depends on task context, sensor setup, and validation conditions.

## Data Availability

No new data were created or analyzed in this study. Data sharing is not applicable to this article.

## Abbreviations

The following abbreviations are used in this manuscript:

ANS	Autonomic Nervous System
BCI	Brain–Computer Interface
CL	Cognitive Load
CLT	Cognitive Load Theory
EDA	Electrodermal Activity
EEG	Electroencephalography
ECG	Electrocardiography
EMG	Electromyography
fMRI	Functional Magnetic Resonance Imaging
fNIRS	Functional Near-Infrared Spectroscopy
Oxy-Hb	Oxygenated Hemoglobin
Deoxy-Hb	Deoxygenated Hemoglobin
HR	Heart Rate
HRC	Human–Robot Collaboration
HRV	Heart Rate Variability
LF/HF	Low-Frequency/High-Frequency Ratio
MHMM	Multimodal Hidden Markov Model
MWL	Mental Workload
NASA-TLX	NASA Task Load Index
PFC	Prefrontal Cortex
pNN50	Proportion of Normal-to-Normal Intervals Exceeding 50 ms
PPG	Photoplethysmography
RMSSD	Root Mean Square of Successive Differences
SCR	Skin Conductance Response
SCL	Skin Conductance Level
